# Luxation postérieure de l’épaule chez un sujet sportif traitée par transfert pédiculé du sub-scapulaire

**DOI:** 10.11604/pamj.2015.22.353.8263

**Published:** 2015-12-11

**Authors:** Adil El Alaoui, Ilyas Rabhi, Aliou Bah, Mouhcine Sbiyaa, Amine Mezzani, Badr Alami, Amine Marzouki, Fawzi Boutayeb

**Affiliations:** 1 Service de Chirurgie Orthopédique du Centre Hospitalier de Chambéry, France; 2 Service de Chirurgie Orthopédique et Traumatologique A, CHU Hassan II, Fès, Maroc

**Keywords:** Luxation, postérieure, épaule, Dislocation, posterior, shoulder

## Abstract

La luxation postérieure de l’épaule constitue une lésion rare et un piège diagnostique et mettant en jeu le pronostic fonctionnel de l'articulation de l’épaule. Les signes cliniques et radiologiques des luxations postérieures de l’épaule ont été bien définis dans la littérature par Bernageau et Patte, mais elles passent le plus souvent inaperçues. Nous rapportons un cas de luxation postérieure de l’épaule chez un sujet sportif de 22 ans pratiquant le Hand-Ball, traité par la technique de transfert pédiculé du sub-scapulaire.

## Introduction

Les luxations postérieures de l’épaule sont des lésions relativement rares, puisqu'elles représentent 5% des luxations de l’épaule. Les lésions anatomiques postérieures peuvent se présenter sous forme de lésions labrales isolées ou associées d'une distension capsulaire ou de lésions osseuses à type de fracture ou érosion de la glène postérieure ou de lésion céphalique de Mac Laughlin [1]. Le traitement chirurgical fait appel à des gestes osseux (butée, ostéotomie de dérotation) ou musculaire (transfert du muscle sub-scapulaire). Nous rapportons l'observation d'une luxation postérieure de l’épaule chez un sujet sportif traité par la technique de transfert du sub-scapulaire.

## Patient et observation

**Figure 1:**
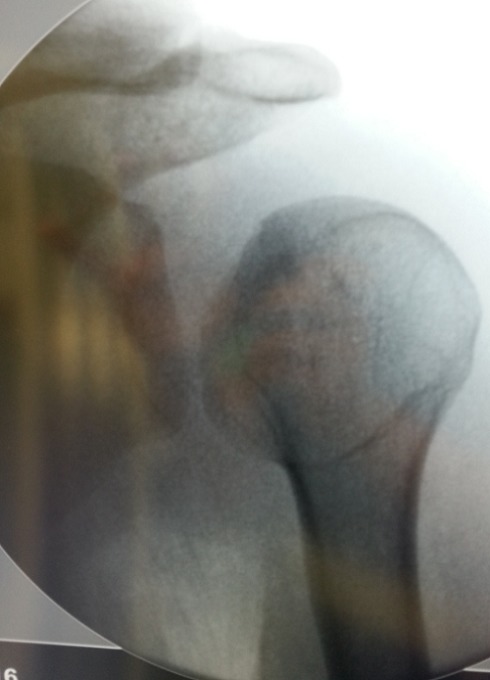
Image scopique de face montrant une luxation postérieure de l’épaule gauche

**Figure 2:**
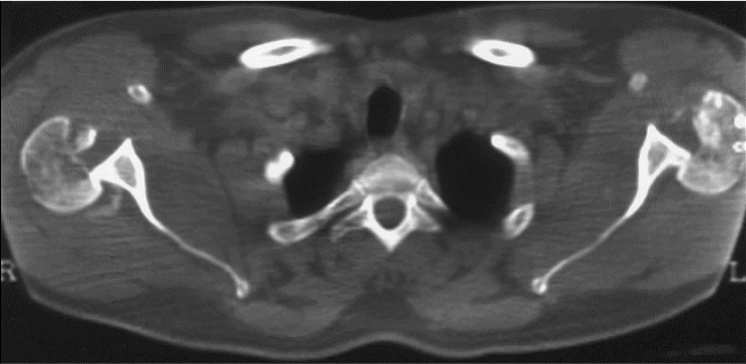
Coupe scannographique objectivant une luxation postérieure de l’épaule avec une encoche cépahalique antérieure

**Figure 3:**
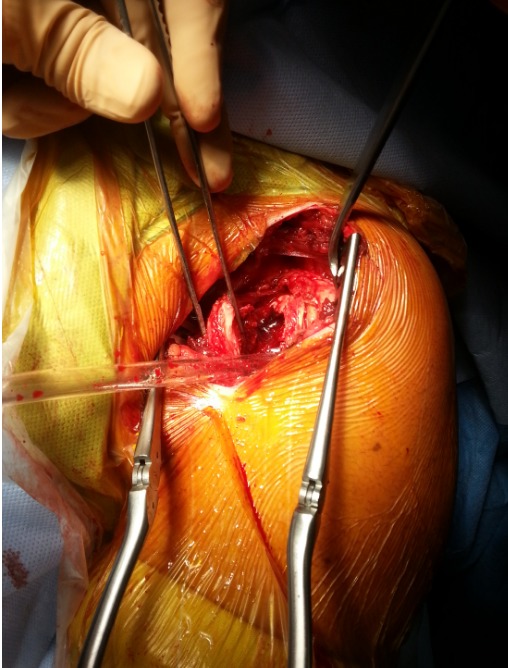
Image peropératoire montrant une encoche importante au niveau la tête humérale après une luxation postérieure de l’épaule

**Figure 4:**
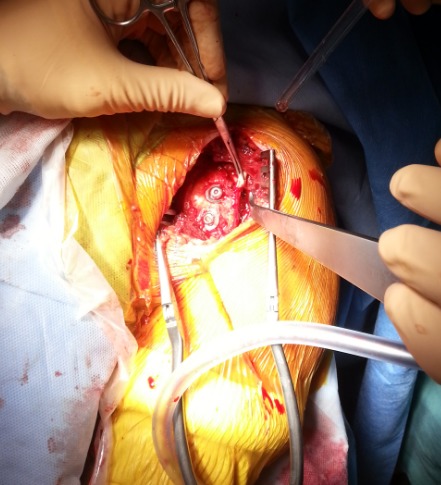
Image peropératoire montrant la fixation par 2 vis du trochin au niveau de l'encoche de Mac Laughlin

**Figure 5:**
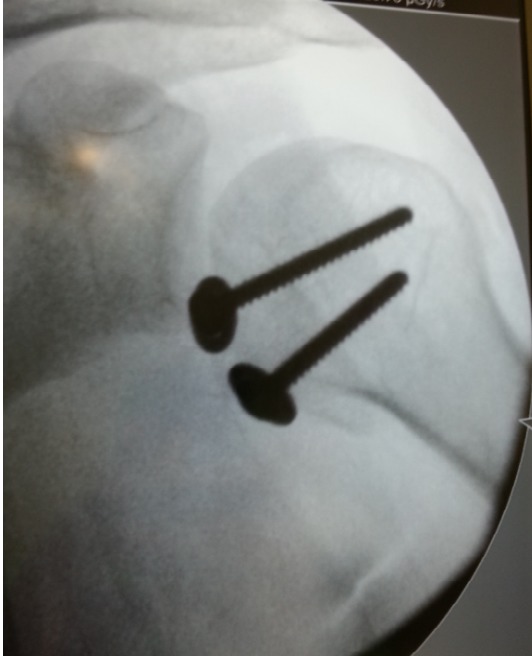
Image scopique post opératoire de l’épaule montrant la réduction de la luxation après stabilisation par 2 vis

Il s'agit d'un patient de 22ans, joueur de hand-ball depuis 7ans, qui a subi un traumatisme lors de la pratique sportive en chutant sur son épaule gauche. L'examen initial a retrouvé une douleur et impotence fonctionnelle du membre supérieure gauche, une épaule bloquée en adduction rotation interne. L'examen vasculo-nerveux est sans anomalies ainsi l'examen cutané. La radiographie de l’épaule face (Figure 1) et profil pratiquées en urgence ont montré une luxation postérieure de l’épaule. Un complément scannographique a été réalisé et qui a objectivé une encoche antérieure de Mac Laughlin de la tête humérale (Figure 2). Une réduction de la luxation à foyer fermé a été réalisée sous anesthésie générale et curarisation, l’épaule restait instable, d'où l'indication d'une réduction à foyer ouvert et stabilisation de l’épaule par transfert musculaire. Cette technique chirurgicale consiste par voie d'abord délto-pectorale à un transfert du muscle sub-scapulaire resté pédiculé sur le tubercule mineure, ce qui offre l'avantage d'un meilleur comblement de l'encoche céphalique par le trochin (Figure 3), et d'une fixation plus solide de la réinsertion du sub-scapulaire par 2 vis spongieuses (Figure 4). Les examens clinique et radiologique de l’épaule en per opératoire montrent une épaule stable avec un secteur de mobilité normal (Figure 5). Un scanner de contrôle réalisé le lendemain de l'intervention qui a objectivé une tête humérale en place, avec un bon comblement de l'encoche céphalique. Le membre supérieur était immobilisé en légère rotation externe pour une durée de 4 semaines suivi de rééducation. La reprise de l'activité sportive était sans problème au 6^éme^ mois.

## Discussion

Le traitement chirurgical des luxations postérieures de l’épaule par transfert musculaire est plus favorable, elle consiste au transfert du sous scapulaire avec comblement de l'encoche par le trochin ce qui oppose la surface arrondie ostéo-articulaire au bord postérieur de la glène lors de la rotation interne et évite aussi l'avalement de l'encoche lors de ce mouvement [2, 3]. Ce transfert du sous scapulaire bride la rotation interne par tension du muscle qui se coude sur le bord antérieur de la glène lors du mouvement luxant. Concernant la voie d'abord, Dubousset, Vichard [4]. abordent ces épaules par voie postérieure pour réparer le décollement capsulo-ligamentaire postérieure. A la suite de A. Trillat et comme Gerber, Hawkins, Rowe, Walch utilisent la voie d'abord antérieure délto-péctorale. [5]. Hawkins et Gerber proposent, plus volontiers, dans les fractures à encoche très volumineuse une arthroplastie d'emblée de l’épaule [5, 6]. nous ne l’ avons pas pratiquée dans ce type de luxation postérieure,d'autant qu’ il s'agissait d'un patient jeune et sportif. Pour les encoches moins volumineuses, le comblement peut aussi être réalisé par d'autres techniques. Dubousset [7, 8] propose un greffon iliaque mis par voie postérieure. Gerber réalise un décabossage du cartilage articulaire et le remplissage du vide sous chondral par l'os spongieux.

## Conclusion

Le traitement chirurgical des luxations postérieures de l’épaule par transfert musculaire du sub-scapulaire nous parait une méthode fiable et reproductible. la mobilisation post-opératoire de l’épaule doit être progressive et surveillée par un kinésithérapeute.
